# Supplementary material for: Chromothripsis during telomere crisis is independent of NHEJ, and consistent with a replicative origin
Source: Genome Res. 2019 May;29(5):737–49. doi: 10.1101/gr.240705.118 (PMC6499312; doi:10.1101/gr.240705.118)
Supplement: Supplemental Material [file supp_gr.240705.118_Supplemental_file_1.zip › contigs/annotated_contigs/DB52/contig.3.DB52_length_550_mean_cov_6.80727272727.docx]

**DB52_length_550_mean_cov_6.80727272727**

```
GGATAAATAAGAGTGAGAAGAAATTTTCTGCTCCCTACATTTTGAAATTTTAATTAAAATGTCCCAGCTGATCTATCTGCTAATGATTA
 >chr4:58243468-58243716 - E=1e-134
TTTGAACAATGATGCAGAGAATCCTTTTTCTCAAAAGAATGAACATCTTTTAAGCCAAGAACAGCTTTGGGTCCAGATGTTACTGTTGT

TCATTCTCACCATTAATCACCAAACATTGCAGTAGGACCCACCATGGATATTATTCAAACAGAGACTCAA|ACTGCTTTCTCTAAGGAT
                                                                        >chr4:58242720-5
CTCAAGTGGGATAATAAAATCTTATTTGGAAACCCTTTCCTAATCATCTCTGCATTAGTCAGGGTTTTACAGAGAATAGAGAAGAGAT|
8242840 - E=1e-60 p=0e+00
TATATATATATATA|ATTTCCTATAGAGAGATATATTTTCCAACTATCTATCTTTATCTATATATGTGTTATATATACATATTATACAT
>chr4:58242502-58242698 - E=3e-100
ATACATATATAGAGATGGAGAGAAAAGCAGAGAGAGAGAGAGGAAGAGAAATACACAGAGCAAGAGAGAGAGATTCTTTTAAAATAAGA

AATTGGCTCACAGGATGAT
```
